# 射波刀治疗31例晚期非小细胞肺癌患者的疗效观察

**DOI:** 10.3779/j.issn.1009-3419.2011.04.05

**Published:** 2011-04-20

**Authors:** 艳玲 吕, 振 王, 锡旭 朱, 泽天 沈, 冬梅 袁, 小辉 缪, 毅 施, 勇 宋

**Affiliations:** 1 210002 南京，南方医科大学南京临床学院，南京军区南京总医院呼吸内科 Department of Respiratory Disease, Nanjing Clinical school of Southern Medical University, Nanjing General Hospital Of Nanjing Military Command, Nanjing 210002, China; 2 210002 南京，南京大学医学临床学院，南京军区南京总医院呼吸内科 Department of Respiratory Medicine, Nanjing University School of Medicine, Nanjing General Hospital Of Nanjing Military Command, Nanjing 210002, China; 3 210002 南京，南京大学医学临床学院，南京军区南京总医院放疗科 Department of Radiotherapy Center, Nanjing University School of Medicine, Nanjing General Hospital Of Nanjing Military Command, Nanjing 210002, China

**Keywords:** 射波刀, 立体定向放射治疗, 肺肿瘤, Cyberknife, Stereotactic radiotherapy, Lung neoplasms

## Abstract

**背景与目的:**

射波刀是近年来新出现的一种立体定向放射治疗技术，具有大剂量、高精度和周围受照射的正常组织或是重要器官的范围小等优点，在治疗非小细胞肺癌（non-small cell lung cancer, NSCLC）方面取得了显著的效果。本研究旨在探讨射波刀（Cyberknife）治疗晚期NSCLC的疗效和安全性。

**方法:**

在2009年3月-2010年3月间，我们应用射波刀治疗31例（34个肿瘤病灶）临床Ⅲ期-Ⅳ期的周围型NSCLC患者，其中Ⅲ期5例，Ⅳ期26例，腺癌15例，鳞癌12例，大细胞癌1例，腺鳞癌2例，不确定NSCLC 1例。28例患者联合了化疗。平均肿瘤体积67.2 cm^3^，处方总剂量36 Gy-60 Gy，分割2次-5次。等计量线65%-85%。治疗后1-2个月复查胸部CT，观察近期疗效，每3个月随访一次。

**结果:**

2例完全缓解，16例部分缓解，7例稳定，6例进展，总体有效率达58%，疾病控制率为81%。所有患者治疗耐受性良好，最主要的不良反应表现为乏力，无4级或是4级以上的不良反应发生。

**结论:**

射波刀治疗晚期NSCLC安全性好，有较好的近期疗效，不良反应轻，患者易耐受。但是远期疗效有待进一步随访。

## Abstract (en)

**Background and objective:**

Recently, Cyberknife is a new flame-less stereotactic radiation therapy technology, which has several advantages, such as large dose, high precision and minimizing exposure to the surrounding normal tissue or adjacent vital structures, so it is successful in the treatment of non-small cell lung cancer (NSCLC). The aim of this study is to determine the effectiveness and safety of robotic stereotactic radiotherapy-Cyberknife with image guidance and realtime respiratory tracking against clinical stage Ⅲ-Ⅳ peripheral NSCLC.

**Methods:**

A review of treatment details and outcomes for 31 patients, with 34 tumors with histologically proven cancers treated by Cyberknife at the CyberKnife Center of Nanjing general hospital of Nanjing military command between March 2009 and March 2010 is presented. Of the 31patients, 15 were adenocarcinoma and 12 were squamous cell cancer. Twenty-eight patients received other forms of antineoplastic therapy such as chemotherapy. A total dose of 36 Gy-60 Gy was prescribed to the 65%-85% isodose line and given in two to fve fractions in less than 1 week using the CyberKnife radiosurgery system. CT scans were performed afer one-two months, then patients were followed every 3 months.

**Results:**

Two patients had complete radiographic responses, 16 patients showed PRs, 7 patients showed SDs. Two patients showed PRs (reduction in tumor size), but developed distant metastases. Response rate was 58% and disease control rate was 81%. All patients tolerated the radiosurgery well, fatigue being the main side effect. No grade 4 or above toxicity was encountered.

**Conclusion:**

In this small cohort of patients with advanced peripheral NSCLC, Cyberknife seems to be a safe and has good therapeutic effects with slight adverse reaction, but long time follow-up is necessary to evaluate the survival data and late toxicity.

放疗是中晚期非小细胞肺癌（non-small cell lung cancer, NSCLC）的主要治疗手段，与化疗同等重要。但常规放疗的疗效并不满意，5年生存率仅为4%-10%^[1]^，即使用三维适型放疗技术，由于肿瘤的放射剂量受周围正常组织的剂量限制而无法进一步提高，局部未控制和复发仍是治疗失败的主要原因。近年来，有研究^[2-4]^结果显示，低分割大剂量的立体定向放射治疗能够克服常规放射治疗的限制，提高局部控制率。射波刀（Cyberknife）是近年来新出现的一种立体定向放射治疗技术，采用实时图像引导系统以及呼吸追踪系统，可作低分次、大剂量、动态的照射，主动跟踪肿瘤，能准确计算肿瘤的运动位置，定位更精确。由于适形性和治疗精度进一步提高，给予肿瘤致死的放射剂量的同时使周围正常组织或是重要器官受照射的体积小，副作用少，安全性好。Whyte等^[5]^在2003年首次用于肺部肿瘤的治疗，并取得了较好的疗效。我院自2009年3月-2010年3月采用射波刀对31例晚期NSCLC患者进行治疗，现将治疗结果报道如下。

## 1 材料与方法

### 1.1 病历资料

选自2009年3月-2010年3月在我院接受射波刀治疗的31例（34个肿瘤病灶）资料完整的Ⅲ期-Ⅳ期NSCLC患者。所有患者均经细胞学或组织学确诊，体能状态评分在0分-3分，其中男性25例，女性6例，年龄38岁-85岁。鳞癌13例，腺癌14例，大细胞肺癌1例，腺鳞癌2例，不确定NSCLC 1例（表 1）。所有的肺部肿瘤均位于肺叶周边，主支气管外2 cm。所有患者中骨转移12例，脑转移4例，同时存在骨转移和脑转移2例，胸膜转移4例，肺转移5例，其它部位转移2例。3例患者针对两个不同病灶先后接受2次射波刀治疗。2例患者因年龄较大，体能状态评分2分-3分，肺功能较差，且合并其他内科疾病，不能耐受化疗以及常规放射治疗，在射波刀治疗前未行任何抗肿瘤治疗。射波刀治疗前，28例患者进行4个-6个周期含铂两药联合化疗方案的治疗。

**1:** 患者以及肿瘤的一般特性 Patient and tumor characteristics

Characteristic	*n*	Patients in stage Ⅲa Patients in stage Ⅲb		Patients in stage Ⅳ
Age (year)				
38-49	6	0	0	6
50-59	8	0	1	7
60-69	3	1	1	1
70-79	10	1	1	8
80-85	4	1	0	3
Gender (*n*)				
Male	25	3	3	19
Female	6	0	0	6
Histology				
Adenocarcinoma	15	1	0	14
Squamous cell carcinoma	12	1	2	9
Large cell carcinoma	1	0	0	1
Adenosquamous carcinoma	2	1	0	1
Undifferentiated carcinoma	1	0	1	0
Tumor diameter (cm)^*^				
1-5	24	2	1	21
> 5	10	1	2	7
Tumor Location^*^				
Left upper lobe	3	0	0	3
Left lower lobe	5	0	0	5
Right upper lobe	15	3	1	11
Right lower lobe	11	0	2	9
Performance score				
0	11	1	1	9
1	11	1	0	10
2-3	9	1	1	7
^*^The number of tumor				

在接受治疗前，每个患者均完成相关检查，包括头颅MRI，胸部以及腹部CT，肺功能，心电图，血常规，血生化，肿瘤标记物，经呼吸科肺部肿瘤医生和放疗科医生对其病情进行评估，并签署射波刀治疗的知情同意书。

### 1.2 定位及扫描

治疗前，采用真空袋将患者按照治疗体位固定，所有患者经德国Siemens产Sensation 16 PET/CT机行CT扫描，扫描时患者屏气，扫描层厚1 mm，扫描范围包括全肺和病灶上下15 cm范围。通过DICOM协议将CT图像传输至图像融合及轮廓勾画工作站（InView^®^，版本1.6.0）。

### 1.3 靶区勾画及剂量要求

在肺窗上勾画靶区，根据肿瘤的体积确定GTV，GTV在x、y、z轴方向各外放1 mm-2 mm形成PGTV。CT V定义为GTV+8 mm，PT V定义为CTV+2 mm-3 mm。要求95%以上PGTV接受60 Gy的照射剂量。将图像及勾画好的靶体积通过DICOM RT协议推至射波刀的SGI工作站（CyberKnife^®^ System，版本7.0.0）。

### 1.4 治疗方法

24例患者行CT引导下经皮肺穿刺金标植入术，置入1枚金标于肿瘤内部或是周边，然后CT扫描观察有无气胸以及出血的表现。7天后进行射波刀治疗。采用红光追踪呼吸运动，记录患者呼吸运动的频率和深度，配合一个呼吸周期内多个时点X光对靶区周围置入金标的移动，以掌握呼吸周期中靶区的内运动模式，随靶区运动做动态的放射治疗。9例患者采用X Lung追踪系统，在治疗计划制订时以CT中心来确认感兴趣区域，软件分析ROI骨性标记，分析病患体位6维误差，使用自动治疗床进行患者定位，在治疗期间，机械臂自动修正位置和方向补偿定位误差。根据患者一般情况、体能状态评分、基础疾病、肿瘤大小以及肿瘤的部位设定剂量分割和每次照射剂量，采用低分割照射（9-20）Gy/次，分割2次-5次，1次/天（周六、周日休息），总剂量36 Gy -60 Gy，治疗时间2天-5天。（剂量学指标详见表 2）

**2:** 射波刀治疗31例晚期NSCLC的剂量学指标 Treatment characteristics of cyberknife for 31 patients with advanced NSCLC

	Total dose (Gy)	Fraction (*n*)	BED (Gy)	Maximum dose (Gy)	Minimum dose (Gy)	Isodose line (%)	Tumor volume coverage (%)
Range	36-60	2-5	83-180	47-83	25-58	65-85	78-100
Mean	48	-	115	64	38	-	-
Median	-	5	-	-	-	77	92
BED: biological effective dose.							

### 1.5 随访以及评价标准

射波刀治疗后4周-12周行CT扫描评价近期治疗效果，此后每3个月随访1次，随访时间1个月-12个月。我们评价的终点是肿瘤局部控制和疾病进展。病灶近期的疗效按照RECIST标准评价，放射损伤按照RTOG放射性肺损伤分级标准评价。

## 2 结果

### 2.1 近期疗效

经射波刀治疗的31例晚期NSCLC患者，4周-12周后复查胸部CT，2例患者达到完全缓解（complete response, CR），16例部分缓解（partial response, PR），7例稳定（stable disease, SD），6例疾病进展（progressive disease, PD）（图 1）。2例病情进展的患者病灶本身缩小达到PR，但是出现了远处转移或是转移灶增大。2例患者出现局部复发。治疗的总体有效率为58%，疾病控制率达81%。亚组分析中显示：生物等效剂量（biological effective dose, BED）≥100 Gy组与 < 100 Gy组相比，得到了更好的局部控制率（84.6%, 60%）和有效率（61.5%, 40%）。处方剂量≥45 Gy组的局部控制率和有效率（63%, 88%）比 < 45 Gy（45%, 57%）组高（表 3）。

**1:**
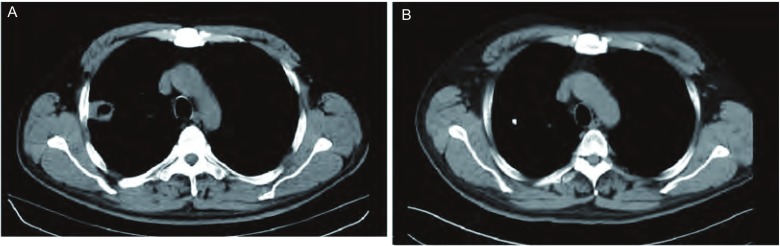
射波刀治疗前和治疗后2个月的胸部CT比较。A：射波刀治疗前的胸部CT；B：射波刀治疗后2个月的胸部CT。 Comparison chest CT before CyberKnife treatment with that at 2 months after treatment. A: Chest CT before CyberKnife treatment; B: Chest CT at 2 months after treatment.

**3:** 射波刀治疗31例晚期NSCLC的近期疗效以及死亡情况 Outcome and death of cyberknife for 31 patients with advanced NSCLC

Subgroup	*n*	RR (%)	DCR (%)	Death (%)
Total	31	58% (18/31)	81% (25/31)	19% (6/31)
Histology				
Squamous cell carcinoma	12	58% (7/12)	75% (9/12)	17% (2/12)
Adenocarcinoma	15	47% (7/15)	80% (12/15)	27% (4/15)
Others	4	100% (4/4)	100% (4/4)	0
Gender				
Male	25	64% (16/25)	80% (20/25)	20% (5/25)
Female	6	33% (2/6)	83% (5/6)	17% (1/6)
BED (Gy)				
< 100	5	40% (2/5)	60% (3/5)	40% (2/5)
≥ 100	26	61.5% (16/26)	84.6 (22/26)	16% (4/26)
Total dose (Gy)				
< 45	7	43% (3/7)	57% (4/7)	28% (2/7)
≥ 45	24	63% (15/24)	88% (21/24)	17% (4/24)
RR: response rate; DCR: disease control rate.				

### 2.2 不良反应

所有的患者治疗耐受性良好，均完成了射波刀的治疗。没有发生4级或5级的严重不良反应。所有患者中，体重均未见明显的变化，乏力是最常见的不良反应。1例患者针对肺部的不同部位的肿瘤，先后接受两次射波刀的治疗，出现3级放射性肺炎，经对症治疗后好转。1例患者出现2级放射性肺炎，2例患者出现1级放射性肺炎，均不需要药物治疗。1例患者在治疗后1个月出现发热、严重的呼吸困难，需要住院治疗。

### 2.3 死亡情况

随访过程中，接受射波刀治疗的31例患者，死亡6例，其中1例患者死于呼吸衰竭，其余5例均死于肿瘤进展。有3例患者在射波刀治疗前体能状态评分为2分-3分。6例死亡患者中3例在治疗后达到PR，1例为病灶稳定，2例出现新病灶。没有患者发生治疗相关的死亡。

## 3 讨论

肺癌是全球癌症死亡的首要原因，严重威胁人类健康^[6]^。NSCLC占全部肺癌的80%，超过三分之二的患者就诊时年龄 > 65岁。由于早期肺癌患者很少有症状，且常规筛查没有受到重视，所以大多数患者确诊时已经是晚期或是发生转移，治疗时比较困难^[7]^。并且多数早期的患者最终都发展成肿瘤进展。此时，大多数患者因为长期吸烟、肺功能很差，或是年龄较大、体能状态评分差、全身重要脏器功能状态差，加上其他内科疾病，多数患者不能耐受化疗以及常规放疗或三维适型放疗；也有一部分患者因多次化疗后对治疗不敏感，局部病灶控制不佳；多数局部晚期NSCLC的患者经化疗和常规放疗后曾经治疗有效但是后来局部复发。射波刀的出现为解决这一难题提供了可行的手段。

射波刀兼具放射外科和放射治疗两种功能，采用比门控技术或是呼吸抑制技术更加先进的同步呼吸追踪定位系统，根据红外摄像机拍摄的呼吸运动周期和影像定位系统拍摄的肿瘤运动轨迹，建立肿瘤随呼吸运动的空间位置模型，并在整个治疗过程中进行比对验证，静态和动态照射中重复定位和瞬时修正照射方向，主动追踪肿瘤。定位更加精确，减少了因呼吸运动而造成的计划靶区体积的增加，大大提高了治疗精准度以及放射生物效应，明显提高了疾病控制率。而且治疗时间短，正常组织受照射的范围小，副作用小，患者耐受性好。相对于X刀、γ刀，射波刀治疗无需框架，而且定位更精确，大大提高了治疗精准度以及放射生物效应。与常规放射治疗相比，照射次数大为减少，每次照射剂量明显增加，突破了常规放疗等中心照射的限制，又有1, 200个照射方向的选择，射束粗细也有12种直径的变化，因此适型指数以及均匀指数通常都比常规放疗为优。与三维适型放射治疗相比，平均照射剂量提高了75%，平均最小照射剂量提高了51%，大幅提高了生物剂量，而正常组织受照射的范围小^[8]^。进一步提高了适形性和治疗精度，平均误差仅为（0.7±0.3）mm，治疗的精准度可达到（0.3±0.1）mm^[9]^。国外相关文献^[10-12]^报道，射波刀在治疗早期周围型NSCLC时取得了显著疗效，局部控制率可达到95%-100%。甚至可能达到与手术一样的治疗效果。

我院在南京地区乃至全国较早的开展了射波刀的治疗，并采用射波刀治疗晚期NSCLC患者。本项研究为探索性研究，目前国内尚无相关文献的报道。纳入31例患者，其中2例达到CR，16例达到PR，7例SD，6例PD的患者中2例病灶本身缩小达到了PR，但是却出现了远处转移或是转移灶增大。仅2例患者出现局部的复发。射波刀治疗晚期NSCLC患者取得了较好的近期疗效。总有效率达到58%，疾病控制率达到81%。但是未达到治疗早期NSCLC患者的疗效。因本项研究中，所有病例均为临床Ⅲ期、Ⅳ期的患者，以Ⅳ期为主，故随访过程中出现远处转移或是转移灶增大的患者较多见。此外，60%的患者年龄在60岁以上，因长期吸烟，肺功能较差，一般情况较差，多数患者曾接受多次化疗，局部病灶未控制，而选择联合射波刀的治疗。为兼顾全身情况，我们没有给予根治性的照射剂量，仅给予姑息治疗。本项研究中，接受射波刀治疗的患者中，肺部肿瘤病灶直径 > 5 cm的患者占30%。有研究^[13, 14]^结果显示，肿瘤大小是影响立体定向放射治疗效果的主要因素。肿瘤越大，局部控制率以及总体生存率越差。此外，本项研究结果发现BED ≥100 Gy组和总剂量≥45 Gy组等到了更好的局部控制率和有效率，说明生物等效剂量和总剂量可能亦是影响放射治疗效果的主要因素，与其他相关研究结果^[14, 15]^相似。然而，射波刀用于治疗肺部肿瘤的单次剂量、总剂量、最大剂量以及最低剂量、分割次数以及间隔时间，目前国内外尚无统一的规定，仍在摸索中。

射波刀提高了肿瘤的放射剂量，而放射性肺炎是限制肺部靶区剂量的主要因素，与照射剂量和范围有关。射波刀高精度的定位以及计划系统，其照射的射束分散、照射精准，能克服患者和肿瘤在治疗过程中的移动。使正常组织被照射的体积减少，同时可以避免敏感组织受到较高剂量的照射，降低放射性肺炎的发生率。本项研究结果显示射波刀治疗后急性放射性肺炎的发生率较低，仅为13%，其他临床研究也得到类似的结果^[16, 17]^。与国外文献^[18]^报道一致，乏力为最常见的不良反应。

本项研究将射波刀用于晚期NSCLC患者的治疗，因为射波刀治疗肺部肿瘤大多数为探索性研究，国内外相关的研究所涉及的样本量均偏少，本研究虽为单中心的研究，但纳入了31例患者，研究结果表明，治疗的近期效果满意，安全性良好。可以为射波刀在国内的广泛应用提供借鉴。但是因病例有限，随访时间较短，远期的疗效、是否有益生存以及晚反应组织损伤的评估有待进一步观察和研究。
